# Functional outcome of surgical management of low mid-grade lumbar spondylolisthesis when considering the sagittal balance parameters preoperatively: a prospective study

**DOI:** 10.1186/s41016-022-00303-2

**Published:** 2022-11-25

**Authors:** Sameh Elmorsy Hassan Elmorsy, Hazem Abdelsattar Abulnasr, Yousry Hassan, Magdy Samra, Ehab Mohamed Eissa

**Affiliations:** 1 Shiekh Zayed Al-nahian Hospital, Cairo, Egypt; 2 Neurosurgery Cairo University, Cairo, Egypt

**Keywords:** Spondylolisthesis, Low-grade spondylolisthesis, Sagittal balance, Lumbar lordosis

## Abstract

**Background:**

**Prospective study objectives**. A sagittal balance is a good tool to improve the functional outcome of spine spondylolisthesis surgeries, primarily noted that it has a good impact in deformity surgery and then applied to every spine surgery and the aim of this study is to evaluate its functional outcome when considered in preoperative planning for non-dysplastic low- and mid-grade spondylolisthesis surgeries.

**Method:**

Forty patients diagnosed as low- or mid-grade non-dysplastic spondylolisthesis had undergone surgery at Cairo University after failed medical treatment had been evaluated preoperatively by measuring the sagittal balance parameters which include SVA, spinopelvic angles, lumbar lordosis, pelvic tilt, sacral slope, and pelvic incidence and then measure it along a follow-up period of 1 year postoperatively started from February 2018 and correlate it with functional outcome using Oswestry score (ODI)and VAS. Correction of parameters has been estimated preoperatively by manual estimation and Surgimap application then applied during the operation.

**Results:**

All patients were treated by surgical treatment through posterior transpedicular screw fixation with conventional or reduction screws and fusion ± TLIF cages. The mean of lumbar lordosis and mean spinopelvic angles were increased in a statistically significant manner. Pelvis tilt was decreased in a statistically insignificant manner. The mean of pelvic incidence was not changed and statistically insignificant, and this is matching the fact that pelvic incidence is a constant parameter. The sacral slope was increased in a statistically insignificant manner.

Final results showed that 37 had a statistically significant improvement in their ODI >20% at the last visit. Three patients had a poor clinical outcome with ODI scorFinal results showed that 37 had a statistically significant improvement in their ODI >20% at the last visit. Three patients had a poor clinical outcome with ODI score of >20% improvement, and we noticed that the level of pathology was at the level of L4L5, SVA was positive and worsen postoperatively, and also, it is accompanied by decreased lumbar lordosis. Change in ODI means statistically significant improvement when considering sagittal parameters preoperation and during operation.

**Conclusion:**

Sagittal balance parameters should be considered in the surgical management of low-grade spondylolisthesis cases to improve their functional outcome.

## Background

Back pain may result from an imbalance in both the coronal or sagittal planes. According to the cone of the economy which has been described by Dubousset (apex is the feet, the base is the head) [1, 2]. The body can stay balanced inside the cone without the need for external support and with minimal effort. When this cone is exceeded body will need much muscular effort to maintain upright and correct this imbalance [1, 2].

Sagittal balance is known to decrease the loads and stresses over the back muscles and to improve the outcome of lumbar spine surgeries especially in cases of spondylolisthesis.

In 2008, Duval-Beaupère’s published a paper entitled the “Sagittal parameters as the most fundamental criteria for understanding the spinal pathologies and their treatment.” [3].

Based on, Roussouly et al. classification for normal spine balance depends on the sacral slope and spinal shape with four types of the normal spine [4, 5]. These recent studies found that the spino-pelvic sagittal alignment has a close relationship to lumbar spondylolisthesis, [6] adult idiopathic scoliosis [7, 8], thoracic angular posterior convex [9], and many other spinal diseases. In addition, it has been proven to be associated with the clinical symptoms and outcomes of these diseases [10, 11].

Patients with unimproved symptoms due to sagittal imbalance, most probably will get benefit from the surgical intervention which involves either spinal instrumentation and fusion with or without osteotomies to restore the spinal alignment.

## Methods

After we got the institutional review board approval, we established a prospective consecutive cohort of patients who had planned to make a surgical intervention for a low-grade spondylolisthesis in (Cairo University Hospital) in the period from February 2018 to July 2018, for either degenerative or isthmic spondylolisthesis.

### Inclusion criteria

Patients with non-dysplastic spondylolisthesis either degenerative or isthmic: (1) age> 30 years, (2) patients with mechanical low back pain and degenerative changes on imaging (degenerative disc disease, facet hypertrophy, degenerative spondylolisthesis); (3) indications for lumbar fusion (failed non-surgical treatment); and (4) patients with low back pain and spondylolisthesis associated with sagittal imbalance after failed adequate medical treatment.

### Exclusion criteria

The exclusion criteria are as follows: (1) patients who had already a complicated surgery of the spine with deficit or infection; (2) patients with incomplete radiological records; (3) history of trauma, tumor, or infection to the spine; and (4) high-grade spondylolisthesis IV or more.

**Fig. 1:**
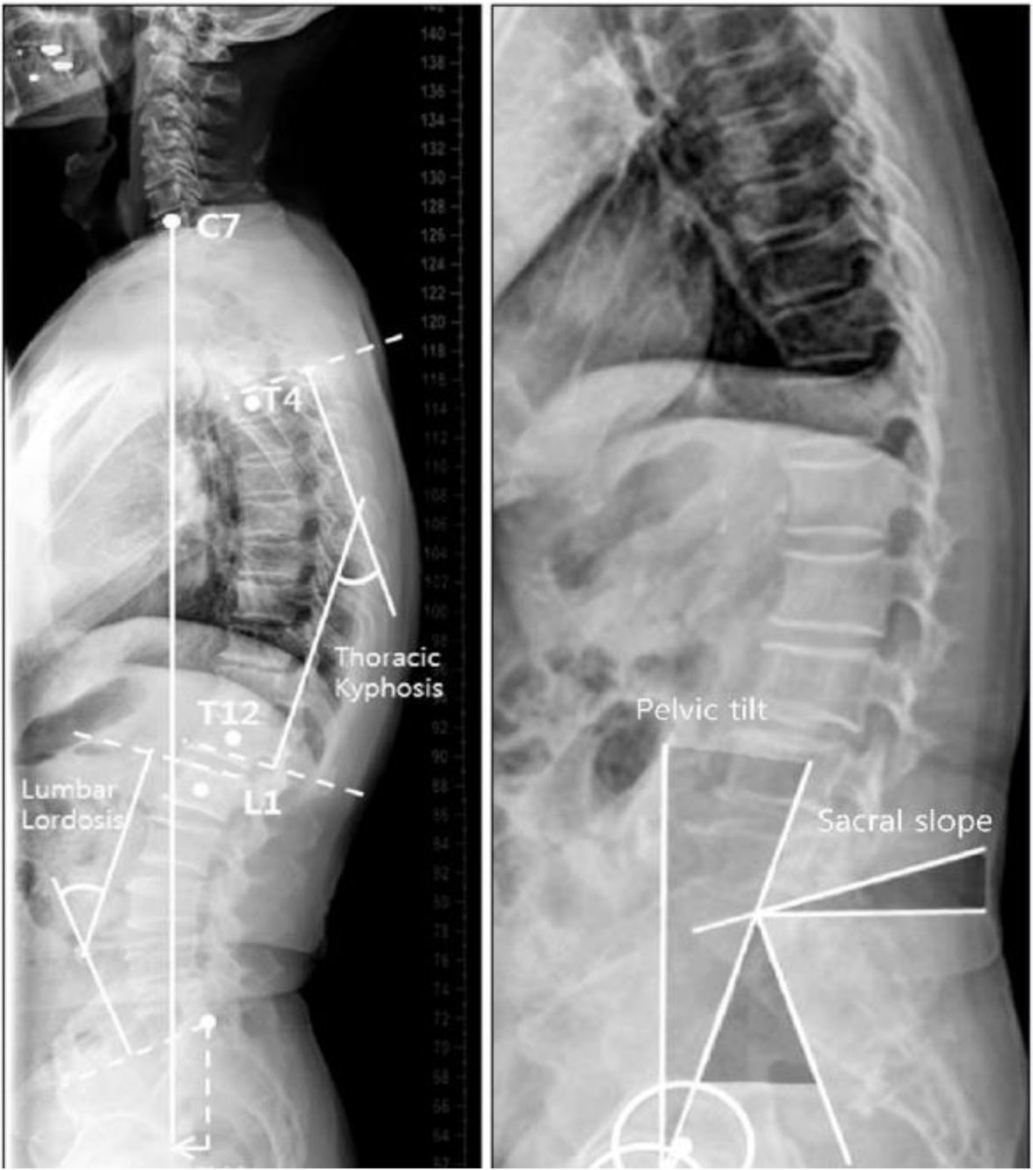
Sagittal balance and spinopelvic parameters

Each patient had a standing anteroposterior and left lateral full spine radiographs including the pelvis, the spinal, and pelvic radiographic parameters had been measured which consisted of PI (pelvic incidence), PT (pelvic tilt), and SS (sacral slope). Spinal parameters include LL (lumbar lordosis) and the number of vertebrae in the lordosis (NVL). Global spinal parameters include SVA (sagittal vertical access) the distance between the C7 plumb line and the posterior superior corner on the top margin of S1 to evaluate the global balance. Degenerative disease of the spine induces local or global kyphosing events, which may be compensated by pelvic retroversion in order to keep the plumb line above the femoral heads (Fig. 1).

### Clinical examination and preoperative preparation

After detailed history and clinical examination were done, the degree of pain according to the patient prescription on the VAS scale was evaluated and his clinical functional ability was evaluated by a preprinted questionnaire of the Arabic version of Oswestry Disability Index (ODI) and then the scoring was estimated, whole spine X-ray to assess SVA on a standard plain X-ray film 24-in. standing lateral view, plain X-ray lumbosacral spine A-P, and lateral position showing both heads of the femurs were requested. After getting radiological films, all radiologic parameters were measured twice by one physician and confirmed later by using Surgimap application for windows version 2.3.2.1. Then, the desired correction was preoperatively determined by the formula described by Schwab et al. LL=PI+9.

### Operative details

All patients were received general anesthesia and treated by using the posterior approach with either posterior transpedicular screws fixation with bending rods to the desired preoperative calculated lumbar lordosis angles with curettage to endplate to induce fusion ± bone graft which was harvested from the patient himself during decompressive laminectomy± usage of TLIF cages on either one level or multiple levels according to the needed degree of correction.

### Surgical technique

We had used the conventional prone positioning for posterior lumbar surgeries, a standard posterior midline conventional incision with subperiosteal dissection of paraspinal muscles till the outer margins of the transverse processes of intended fixation levels were clearly exposed, the facet joints capsules were kept intact. In fixation, we have used the intersection technique in identifying our entry points. After the insertion of screws, full laminectomy was done and foraminotomy of all desired levels. Then, we start preparation for fusion either by decortication of endplates or medial facetectomy to insert TLIF cage using a suitable size of cage which was Zimmer cage size 8–10-mm height. Rod length was taken, and bending rods to the desired lumbar lordosis were done as planned preoperatively.

### Postoperative

Patients were encouraged to be ambulant on surgery night. A standing AP and lateral radiographs were obtained before the patient dismissal from the hospital. The day after surgery patient was evaluated by follow-up plain X-ray lumbar spine lateral view, visual analog scale VAS score, and Oswestry disability index score ODI score and then interpreted. In order for the results to be deemed clinically significant, a change in the patient’s score of 10% or more is required.

### Follow-up

In the first postoperative outpatient clinic visit 10–14 days postoperatively, the patient was evaluated clinically to check the operative site, ambulation, functional outcome, and pain relief. On the second visit of 1–3 months, the patient was asked to do another whole spine plain X-ray film to calculate SVA as preoperative and plain X-ray lumbosacral spine to calculate spinal and spinopelvic parameters by the same way as preoperative. X-ray measurements were used for statistical analysis, and then other visits, the patient was evaluated clinically by VAS and ODI unless other investigations were needed. The other visits of 6 months and at 1 year were to follow functional outcomes and pain relief by VAS and ODI. There was no need for more images in 6 months visit, but all images were repeated at 1-year visit. The follow-up of patients was done for 1 year.

### Statistical analysis

Data collected were reviewed. Coding and statistical analysis of collected data were done by using SPSS program (statistical package of social science; SPSS Inc., Chicago, IL, USA) version 16 for Microsoft Windows.

- Descriptive statistics: The mean and standard deviation (SD) were used to describe quantitative data.
- Analytic statistics: Comparing groups was done using Wilcoxon signed-rank test for comparison of preoperative and postoperative two conditions while Friedman test was used to measure the difference between preoperative and postoperative with various periods. The level of significance was taken at *p* value of <0.05 with a confidence level of 95%. The results were represented in tables and graphs.

## Results

This study included 40 patients with symptomatic lumbar spondylolisthesis with different aetiologies either degenerative or isthmic type. It was designed as a prospective cohort study without randomization to calculate the changes in global and regional sagittal balance parameters and its effects on surgical outcomes.

Initially, we had 48 patients in the study; however, 2 patients with symptomatic back pain and low-grade spondylolisthesis were excluded from this study after we afforded them a chance for adequate nonsurgical treatment again after failed previous treatment by other colleagues, 3 patients did not continue the whole follow-up period, and one patient died during the study due to cardiac problems, and then, we have chosen 40 patients from 42 patients randomly to do our statistics.

**Fig. 2:**
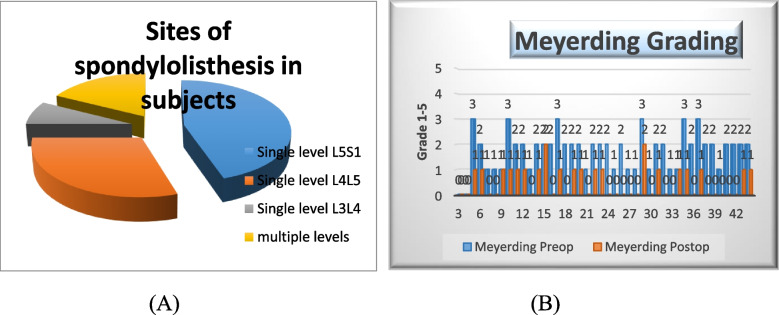
**A** Sites of spondylolisthesis. **B** Preoperative and postoperative grading of study patients

These 40 patients are 17 males and 23 females with mean age = 49.05 years and a range of 33–68 with different aetiologies of spondylolisthesis including 14 degenerative type and 26 isthmic types. It is involving the L3 L4 level in 3 patients, L4 L5 level in 12 patients, L5S1 level in 18 patients, and multiple levels in 7 patients, and 20 patients were grade 1 Meyerding, 14 were grade 2, and 6 patients were close to grade 3 (Fig. 2A).

All patients were treated by surgical treatment through posterior transpedicular screw fixation with conventional or reduction screws and fusion ± TLIF cage insertion. After study of their global sagittal balance by SVA through whole spine X-ray images to choose the most suitable treatment to preserve sagittal balance or to correct the imbalance by a manual method done by one physician twice or by using the Surgimap app for windows V2.3.2.1., and if there is a difference in measurements we took the average, this was needed for a few patients. Fusion was done either by curettage of endplate and impaction of bone grafts to induce fusion or by TLIF cages, and 15 cages were used in 14 patients as one of these patients has needed 2 cage insertion.

All levels are with flat endplates to exclude the dysplastic type. Estimation of the desired correction of angles has been done preoperatively to be close to the normal range of sagittally balanced normal individuals and then were done intraoperatively either by increasing the bending of rods and curettage of endplates to induce fusion with autologous bone graft or by adding TLIF cages to the transpedicular fixation system. None of these patients had a slippage greater than 75% on the Meyerding grading system. The degree of the slippage of vertebrae was improved in all cases either one grade or more on the Meyerding grading scale as shown in Fig. 2B with an average operation time of about 2 h and average intraoperative blood loss of about 400 cc blood.

**Fig. 3:**
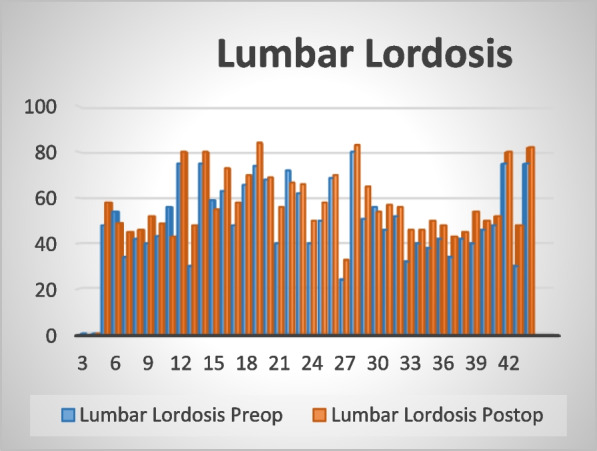
Showing changes in lumbar lordosis postoperatively in comparison to preoperative

The mean sagittal vertical access (SVA) was 4.095 cm preoperatively and 1.31 cm at the last observation correction of lumbar lordosis has an average of 57.93° postoperatively compared to an average of 51.46° preoperatively as shown in Fig. 3. Changes in the mean of spino-pelvic angles, PI, PT, and SS have shown statistically insignificant changes postoperatively compared to preoperative as shown in Table 1.

**Table 1:** Statistical analysis of grading of spondylolisthesis and global and regional sagittal parameters comparing pre with postoperative

	Mean	Std. deviation	*P* value
Pre-Meyerding grading	1.8077	0.69393	0.000*
Post-Meyerding grading	0.62	0.637	
Pre-SVA Cm	3.92	2.226	0.000*
Post-SVA Cm	1.40	2.678	
Pre-Dpinopelvic angle	131.27	10.817	0.008*
Post-Spinopelvic angle	133.81	10.564	
Pre-lumbar lordosis	54.55	15.263	0.002*
Post-lumbar lordosis	60.01	13.505	
Pre-pelvic tilt	22.50	8.793	0.206
Post-pelvic tilt	21.61	6.124	
Pre-sacral slope	42.37	10.699	0.412
Post-sacral slope	43.46	10.573	
Pre-pelvic incidence	64.90	12.591	0.434
Post-pelvic incidence	64.76	11.986	

*Significant difference (*p* value <0.05)

As shown in Table 1, the mean of lumbar lordosis was increased in a statistically significant manner to gain a *P* value of 0.002, preoperatively “54.55 and “60.1” at the final visit. Mean spinopelvic angles increased from “131.27” to “133.81” with a *P* value of 0.008 statistically significant. Pelvis tilt decreased from “22.50” to “21.61” which is statistically insignificant. The mean of pelvic incidence was “64.9” with almost no change to be “64.76” at the final visit which is also statistically insignificant, and this is matching the fact that pelvic incidence is a constant parameter. The sacral slope increased from “42.37” to “43.46” which is statistically insignificance.

**Fig. 4:**
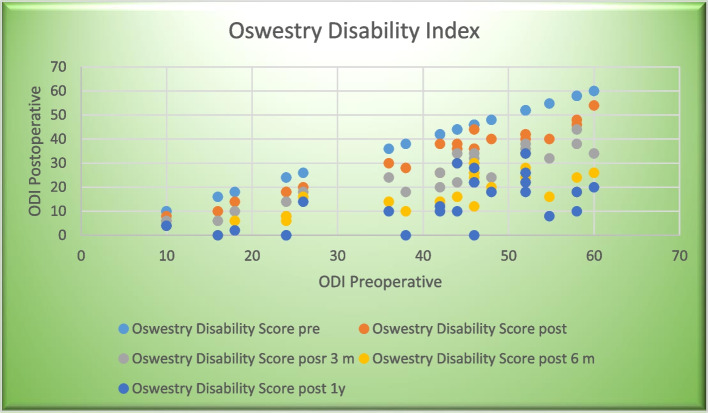
Changes in the mean of ODI during the period of this study

The final follow-up of 40 patients showed that 37 had a statistically significant improvement in their Oswestry score of more than 20% at the last visit. Three patients had a poor clinical outcome with ODI score of less than 20% improvement, and we noticed that the level of pathology was at the level of L4L5 and SVA was positive and worsen postoperatively; also, it is accompanied by decreased lumbar lordosis. Change in ODI is shown in Fig. 4 and Table 2 in which the mean of preoperative is “40.1846” which decreased to be “13.1538” with a *P* value 0.000 which means statistically significant improvement when considering sagittal parameters preoperation and during operation (Fig. 4).

**Table 2:** Changes in mean ODI during the period of this study

	Mean	Std. deviation	*P* value
Pre_Oswestry_Disability_Score	40.1846	15.07071	0.000*
Oswestry_Disability_Score post1	31.8462	13.05892	
Oswestry_Disability_Score post3	24.5385	11.30745	
Oswestry_Disability_Score post6	17.1538	9.26366	
Oswestry_Disability_Score post12	13.1538	10.35642	

Significant difference (*p* value <0.05)

**Fig. 5:**
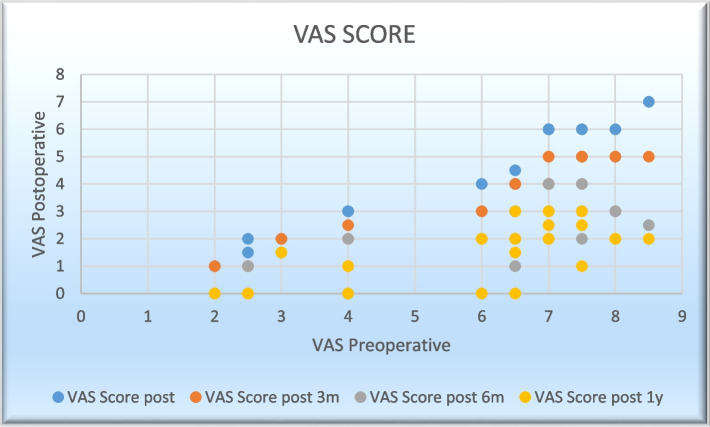
Changes in the mean of VAS scaling during the period of this study

There was also decreased pain score with a mean of “6.0” to reach “1.6154” at the end of the study with a statistically significant decrease in pain when considering sagittal balance parameters during planning for surgery as shown in Table 3 and Fig. 5.

### Complications

Regarding mortality rate in this study was 2.5%. Two reoperations were done due to deep wound infection in patients with chronic diseases, we made revisions without removal of hardware; fortunately, the infection did not reach the fixation system and after drainage of the pus an aggressive treatment after reoperation with suitable antibiotics according to culture and sensitivity patients have healed the operation site well without the need for more interventions in about 1 month and improved in follow-up period to get lesser ODI score by about 10%. The morbidity rate in this study was 7.5% which represents three patients that had poor final outcomes with ODI score of less than 20% improvement.

**Table 3:** Changes in the mean VAS values during the period of this study

	Mean	Std. deviation	*P* value
VAS_pre	6.00	1.908	0.000*
VAS_post1	4.15	1.599	
VAS_post3	3.1346	1.38244	
VAS_post6	2.0192	1.17882	
VAS_post12	1.6154	1.07989	

### Review of selected case

**Fig. 6:**
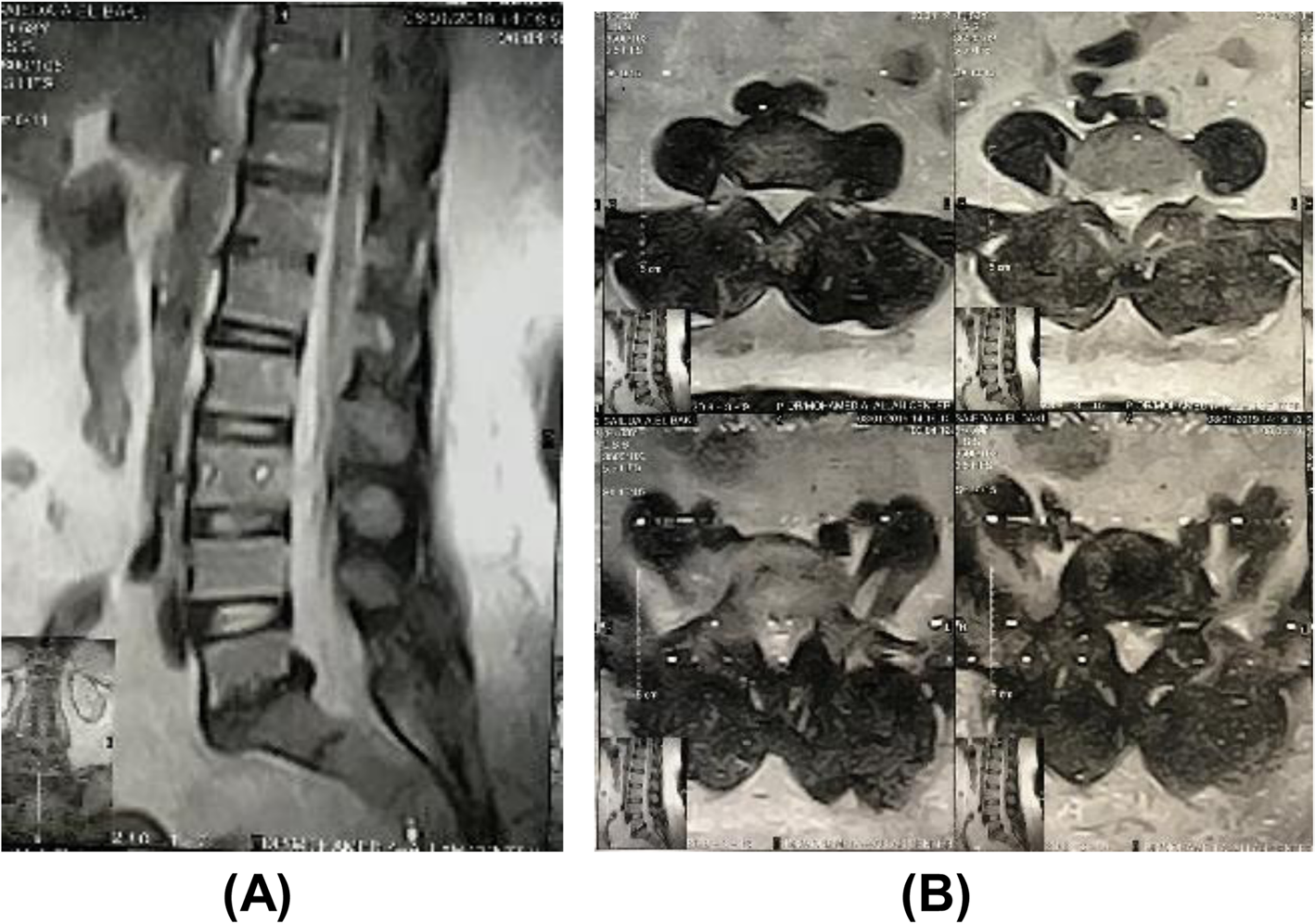
Preoperative MRI scan of the patient **A** sagittal and **B** axial cuts showing lumbar disc prolapse of L5S1 with low-grade spondylolisthesis grade 1

**Fig. 7:**
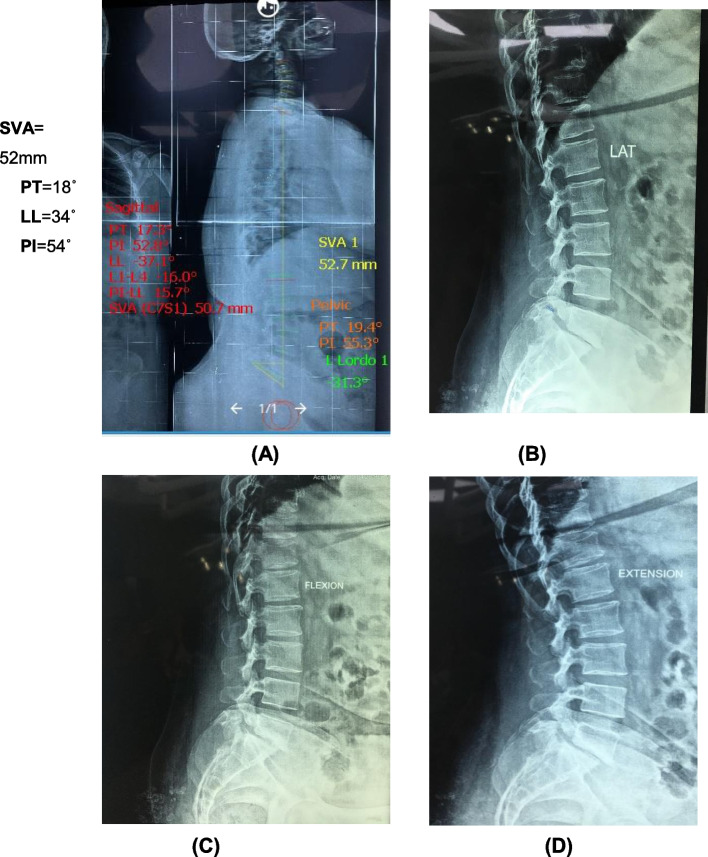
Preoperative X-rays (**A**) shows the calculation of SVA and other pelvic parameters. **B** Preoperative lateral view isthmic spondylolisthesis. **C**, **D** Flexion and extension views

A 62-year-old female patient with a history of lower back pain and bilateral claudication pain for 6 months with a tendency to bend forward to decrease pain and took more than 3 months for adequate medical treatment by examination patient is full motor power with normal tone and reflexes SLR test bilateral 60° ambulant with bending of trunk forward shows her preoperative images with VAS score 6 and ODI 38. Then, MRI lumbar spine, flexion, extension plain X-rays study, and whole spine sagittal balance images were done. In preoperative images, SVA=52 mm, PI=54, PT=18, LL= 34, and Meyerding grading estimated from these images were grade one isthmic spondylolisthesis (Figs. 6 and 7).

The patient was treated by posterior lumbar approach with transpedicular fixation of L4,5, S1+induction of fusion by the harvested bone and curettage of the endplate of L5S1 with pending of rods to increase lumbar lordosis intraoperative correction was obtained without the need to more instrumentation.

**Fig. 8:**
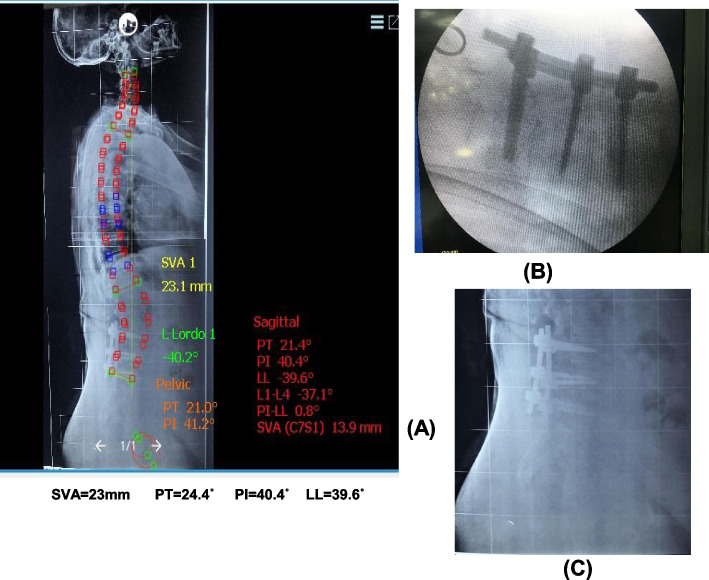
**A** Postoperative X-ray of whole spine shows postoperative parameters of SVA and pelvic parameters. **B** Intraoperative imaging of instrumentation. **C** Lateral X-ray of lumbar spine postoperative

Intraoperative images show a reduction of spondylolisthesis, and postoperative images show changes of SVA=23, PT=24.4, PI=40.4, LL=39.6, and final VAS=0, ODI=0 (Fig. 8).

## Discussion

Most studies that considered the sagittal balance and spinopelvic parameters in literature are retrospective analyses of collected data from follow-up records of registered patients who already have done their surgery without considering these parameters in preoperative planning.

The prevalence of spondylolisthesis in population-based studies have suggested that lumbar spondylolysis have a prevalence of 6% in adult [12, 13]. and that 25% of these populations with spondylolysis experience at least a period of significant back pain in their lifetime.

Isthmic spondylolisthesis appears in a majority of individuals with spondylolysis. Sixty-eight percent of first graders of spondylolysis have been shown to have associated isthmic spondylolisthesis [12]. And this is matching our results as we had 26 patients of isthmic type and 16 of degenerative type, which involved the L3 L4 level in 3 patients, L4 L5 level in 12 patients, the L5S1 level in 18 patients, and multiple levels in 7 patients, with a prevalence of level L5, S1 in the isthmic type and L4,5 in the degenerative type.

So far, there are no definite methods except radiological images to evaluate the instability. So assessment of instability of the spine is a radiological term. Accordingly, the definition of instability is the motion of the above vertebra over expected normal values in the normal spine. In the definition of White and Panjabi, it is the displacement in the sagittal plane of more than 4.5 mm or angulation of more than 22° [14]. Nachemson defined it as translational motion of more than 3 mm and angular motion of more than 10° between L1 and L5 and (more than 4 mm translational motion and 20° angular motion at L5, S1) [15]. In this study, we have considered the Panjabi definition.

Many studies support the improvement of functional outcomes in the different surgical managements of a low-grade spondylolisthesis after the failure of adequate medical treatment and their pain relive either radicular or lower back pain compared to the present study with the same result [16–18]. Recent NASS guidelines have recommended surgical decompression with fusion in cases of degenerative lumbar spondylolisthesis over decompression only or nonsurgical options with better clinical outcomes [19].

Different types of surgery for the management of a low-grade spondylolisthesis have been described in the literature including laminectomy, posterior lumbar intervertebral body fusion (PLIF), transforaminal lumbar interbody fusion (TLIF), anterior lumbar intervertebral body (ALIF), oblique lumbar interbody fusion/anterior to psoas (OLIF/ATP), lateral lumbar interbody fusion (LLIF), and extreme lateral interbody fusion (XLIF). There is no evidence that one surgical approach is clinically superior to another. There is an increasing trend toward MIS approaches due to less intraoperative blood loss; however, long-term data is lacking [20–22].

It is now well accepted that with surgical correction, some spinopelvic parameters of spondylolisthesis such as slip reduction, segmental, and global lumbar lordosis, and some reduction can improve spinopelvic balance and the shape of the lumbar spine but most studies focused on high-grade spondylolisthesis [23–26].

In this study, the preoperative analysis of spinopelvic parameters and the global sagittal balance shows that low PI patients were a small group compared to high PI patients. Low PI reduction of their spondylolisthesis was more correctable than high PI patients, but this was not analyzed in this study because low PI was a small group of patients. Too high pelvic tilt angles showed a postoperative decrease in values of these angles with reduction and fusion, and this provided more physiological value that decreased muscle strain and gave a more favorable outcome. Surgery increased PT to higher values than preoperative which is compatible with the high PI of most of the patients in this study. Although these changes are not statistically significant, mostly due to that there is no major sagittal imbalance noticed in most of our cases as this study included mid and low-grade spondylolisthesis [27].

We have used bending rods± fusion by harvesting bone grafts from the bone that we removed by decompression of theca and nerve roots this gave us the correction of lumbar lordosis with a range of 5–8° in postoperative images. When we need more, we add a PLIF cage which increases our correction to 10–15° in postoperative images. We did not use hyperlordotic cages as it is not available, and our patients did not need more correction than 15°. And this is matching Bourghli et al.’s and Harimaya et al.’s studies [28, 29].

Several studies demonstrated a single pedicle subtraction osteotomy (PSO) can generate 20 to 40° of LL and an approximate 10 to 12 cm change in SVA, depending on the wedge of bone removed [30–34]. Vertebral column resection (VCR) is a procedure of last resort and only considered when more conservative osteotomy will not suffice. Posterior VCR (PVCR) involves resection of all posterior elements, facet joints above/below, pedicles, entire vertebral body, and discs above/below. VCR allows for the tremendous ability to correct the deformity as the entire spine is disarticulated and shortened. In this study, there was no need for osteotomies to increase the degree of sagittal balance correction as bending rods ± TLIF were enough.

Using a short-segment fixation with fusion is the main concept of spine surgeons apart from scoliosis surgeons who prefer long-segment fixation which increases the risk of the flat back syndrome [35]. Unless needed, it is enough for a low-grade lumbar spondylolisthesis to use a short segment of spinal fusion as reported by Cho et al. [36], with great stress on the importance of considering spinal alignment by the restoration of LL in the treatment of a low-grade spondylolisthesis as reported by Lee et al. [37], and loss of LL is postoperatively being associated with increased incidence of low back pain and adjacent segment disease, which was reported in many studies [38–42]. In this study, a short-segment fixation was done in all patients without the need for a long segment which may be because we were working on a low-grade spondylolisthesis.

Our results are matching the results of Jackson et al. who had found the difference in the C7 plumb line to S1 offset between patients with spondylolisthesis and normal individuals [43]. Harroud et al. found a major difference in the sagittal vertical axis between high-grade and low-grade spondylolisthesis [44].

Hresko et al. recommended that partial reduction and instrumentation may be the most important determinant of outcomes, as no correlation was found in his series between the amount of reduction of spondylolisthesis and the improvement in the pelvic tilt [27].

### Functional outcome

Bourghli et al. suggested that the most important point to increase the functional outcome postoperatively is to reposition L5 over S1 as measured by L5 incidence and lumbosacral angle (LSA), rather than reduction of the spondylolisthesis grade. In our study, LSA improved after surgery, moving toward a more normal value, without a statistical significance, but the moderate improvement in LSA showed that L5 repositioning occurred. And this is similar to our results [28].

In this series, the functional outcome is satisfactory with high statistical significance either in pain relief which had tested by VAS scale or gain more improvement in function as tested by ODI score, and this is also the same results in the series of Bourghli et al. [28] and Korovessis et al. [45], but the later one is a retrospective study.

Our results are similar to the results of a meta-analysis done by Kwon et al. in which a better outcome for the treatment of spondylolisthesis is by using instrumented posterior spinal fusion in combination with an interbody graft as opposed to either PSF treatment alone or interbody graft alone [46]. The positive impact of interbody support in the surgical treatment of spondylolisthesis on radiographic and clinical outcomes which has been confirmed by Molinari [47]. This is contrary to the results of Hsu et al. who founded that surgical outcomes in the treatment of a low-grade lumbar degenerative spondylolisthesis with spinal fusion are not correlated with restoration of the LL. And they explained that in their retrospective study, the sagittal balance of patients was in the normal range, preoperatively [48].

The limitations of this study is that it is not a long-term follow-up, and no randomization and there is no control group.

## Conclusion

Surgical management of symptomatic low- and mid-grade nondysplastic spondylolisthesis had shown better functional clinical outcome and more control of pain when considering the restoration of nearly normal values of the pelvic position-dependent parameters and global sagittal balance parameters which are the pelvis tilt, sacral slope, and SVA.

Preservation or correction of lumbar lordosis in accordance with pelvic incidence are important factors that have a positive impact on the functional outcomes of a low-grade spondylolisthesis surgery.

Accurate biomechanics and radiological study of the pelvis and its relation to the spine to assess the impact of the spondylolisthesis on global sagittal balance has a great effect on postoperative outcome.

To correct lumbar lordosis in a low-grade spondylolisthesis in most cases you will need short segment fixation with curved rods + fusion which is better done by TLIF prosthesis. But considering sagittal balance and spinopelvic parameters will guide you on which is the most appropriate surgery for every patient to gain better outcomes and decrease the rate of postoperative failed back syndrome.

## Data Availability

The datasets generated and/or analyzed during the current study are available from the corresponding author on reasonable request.

## Abbreviations

PI: Pelvic incidence
PT: Pelvic tilt
SS: Sacral slope
LL: Lumbar lordosis
NVL: Number of vertebra in the lordosis
SVA: Sagittal vertical access
ODI: Oswestry disability index score
VAS: Visual analog score
SD: Standard deviation
PLIF: Posterior lumbar intervertebral body fusion
TLIF: Transforaminal lumbar interbody fusion
ALIF: Anterior lumbar intervertebral body
OLIF/ATP: Oblique lumbar interbody fusion/anterior to the psoas
LLIF: Lateral lumbar interbody fusion
XLIF: Extreme lateral interbody fusion
PSO: Pedicle subtraction osteotomy
VCR: Vertebral column resection
LSA: Lumbosacral angle
